# Partial Decay of Thiamine Signal Transduction Pathway Alters Growth Properties of *Candida glabrata*

**DOI:** 10.1371/journal.pone.0152042

**Published:** 2016-03-25

**Authors:** Christine L. Iosue, Nicholas Attanasio, Noor F. Shaik, Erin M. Neal, Sarah G. Leone, Brian J. Cali, Michael T. Peel, Amanda M. Grannas, Dennis D. Wykoff

**Affiliations:** 1 Department of Biology, Villanova University, Villanova, Pennsylvania, United States of America; 2 Department of Chemistry, Villanova University, Villanova, Pennsylvania, United States of America; Texas A&M University, UNITED STATES

## Abstract

The phosphorylated form of thiamine (Vitamin B_1_), thiamine pyrophosphate (TPP) is essential for the metabolism of amino acids and carbohydrates in all organisms. Plants and microorganisms, such as yeast, synthesize thiamine *de novo* whereas animals do not. The thiamine signal transduction (THI) pathway in *Saccharomyces cerevisiae* is well characterized. The ~10 genes required for thiamine biosynthesis and uptake are transcriptionally upregulated during thiamine starvation by *THI2*, *THI3*, and *PDC2*. *Candida glabrata*, a human commensal and opportunistic pathogen, is closely related to *S*. *cerevisiae* but is missing half of the biosynthetic pathway, which limits its ability to make thiamine. We investigated the changes to the THI pathway in *C*. *glabrata*, confirming orthologous functions. We found that *C*. *glabrata* is unable to synthesize the pyrimidine subunit of thiamine as well as the thiamine precursor vitamin B_6_. In addition, *THI2* (the gene encoding a transcription factor) is not present in *C*. *glabrata*, indicating a difference in the transcriptional regulation of the pathway. Although the pathway is upregulated by thiamine starvation in both species, *C*. *glabrata* appears to upregulate genes involved in thiamine uptake to a greater extent than *S*. *cerevisiae*. However, the altered regulation of the THI pathway does not alter the concentration of thiamine and its vitamers in the two species as measured by HPLC. Finally, we demonstrate potential consequences to having a partial decay of the THI biosynthetic and regulatory pathway. When the two species are co-cultured, the presence of thiamine allows *C*. *glabrata* to rapidly outcompete *S*. *cerevisiae*, while absence of thiamine allows *S*. *cerevisiae* to outcompete *C*. *glabrata*. This simplification of the THI pathway in *C*. *glabrata* suggests its environment provides thiamine and/or its precursors to cells, whereas *S*. *cerevisiae* is not as reliant on environmental sources of thiamine.

## Introduction

Thiamine, or vitamin B_1_, is composed of two ring structures, a thiazole (4-methyl-5-β-hydroxyethylthiazole, HET) and a pyrimidine (2-methyl-4-amino-5-hydroxymethylpyrimidine, HMP) [1]. Thiamine is pyrophosphorylated, resulting in thiamine pyrophosphate (TPP), which is the active cofactor in amino acid and carbohydrate metabolism [2]. Identified as co-carboxylase, it is required for most *in vivo* decarboxylation reactions. Plants and microorganisms synthesize thiamine *de novo* whereas animals do not [3].

**Fig 1:**
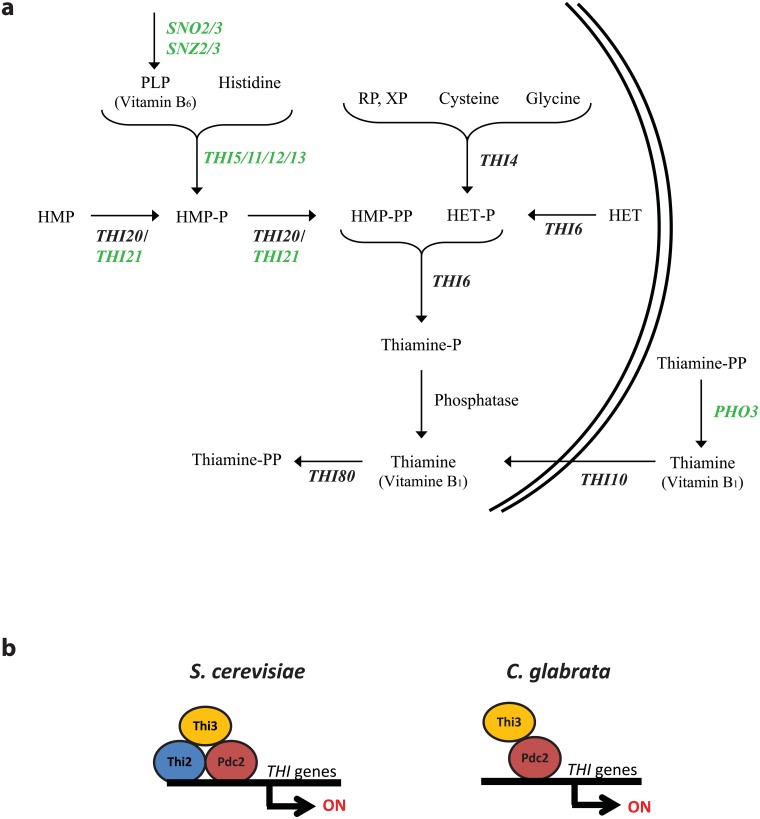
Thiamine biosynthesis pathway and regulation in *S*. *cerevisiae* and *C*. *glabrata*. (A) A schematic of the thiamine biosynthesis pathway with shared components labeled in black and those found only in *S*. *cerevisiae* labeled in green. Lack of Thi21p in *C*. *glabrata* likely does not result in the inability to phosphorylate HMP because Thi20p is present. The schematic is a compilation of data from [4,12]. All compounds are described in the text except RP and XP which are ribulose-5-phosphate and xylulose-5-phosphate (B) A schematic of the transcriptional regulation of THI genes in *S*. *cerevisiae* and *C*. *glabrata*.

*Saccharomyces cerevisiae* synthesizes TPP *de novo* through steps of condensation, hydrolysis, and pyrophosphorylation of HMP-PP and HET-P [4] (Fig 1A). HMP synthesis utilizes the proteins encoded by genes *SNO2*/3 and *SNZ2*/3, which are regulated by thiamine availability, to make the vitamin B_6_ (also known as pyridoxal-5’-phosphate or PLP) [4,5]. Members of the *THI5* gene family (*THI5/11/12/13*) are all functionally redundant and synthesize HMP-P from PLP [6,7]. After being formed, HMP-P is phosphorylated by either Thi20 or Thi21 to form HMP-PP; however, Thi20/Thi21 is also capable of phosphorylating HMP to HMP-P [8]. The single copy *THI4* gene is involved in HET-P synthesis, and during thiamine-depleted conditions, transcription of *THI4* produces one of the most abundant proteins in the cell [9]. Recently, Thi4p was discovered to act as a co-substrate and not an enzyme and can only undergo a single reaction, meaning it is a suicide thiamine thiazole synthase [10]. HET phosphorylation to form HET-P is catalyzed by an enzyme, HET kinase, that is encoded by *THI6* [1]. *THI6* also condenses HMP-PP and HET-P to thiamine phosphate. The final steps of TPP synthesis require the dephosphorylation and pyrophosphorylation of thiamine phosphate. The pyrophosphorylation is carried out by thiamine pyrophosphokinase and is encoded by *THI80* [4,11].

In addition to being able to synthesize thiamine *de novo*, *S*. *cerevisiae* can utilize extracellular thiamine to synthesize TPP. There are two genes that are known to be involved in the acquisition of extracellular thiamine, *THI10* (also known as *THI7*) and *PHO3* [13–15]. *THI10* encodes for a thiamine transporter (and there are likely additional thiamine transporters [16]) in the plasma membrane while *PHO3* encodes a periplasmic acid phosphatase that allows for the intake of thiamine by dephosphorylating TPP to thiamine [4]. Both of these genes are under control of the THI signal transduction pathway and are induced during thiamine-depleted conditions. *S*. *cerevisiae* can also utilize extracellular HMP and HET to form TPP. HMP is thought to be brought into the cells via thiamine transporters but HMP also can be released from yeast cells when too much HMP is present [17]. HET is brought into the cells through diffusion and is trapped due to HET kinase-catalyzed phosphorylation [18].

To prevent wasted energy, when thiamine is present in the environment, thiamine is not biosynthesized and the high-affinity transport system for thiamine (but presumably not the low-affinity transport system) is down regulated [2,19]. Three positive regulatory factors have been identified in the THI regulatory pathway, *THI2*, *THI3*, and *PDC2* [16,19,20]. Both Thi2p and Pdc2p have DNA-binding domains in the N-terminus and appear to interact with Thi3p (Fig 1B). When any of the regulatory factors are deleted, the transcriptional induction of genes in response to low thiamine environmental conditions is dramatically diminished [4].

In our studies with *C*. *glabrata*, we noted that the gene encoding the transcription factor *THI2* is found in *S*. *cerevisiae* but not in *C*. *glabrata*. We also noted that some parts of the THI pathway seem to be lacking in *C*. *glabrata* relative to *S*. *cerevisiae*. *C*. *glabrata* has been demonstrated to be a thiamine and pyridoxine auxotroph previously [21–23] but we expected that the entire pathway would be absent. There are many examples of full pathway degradation in *C*. *glabrata* [24–26], but our interest was piqued by the apparent partial loss of the THI pathway. Additionally, given the evolutionary distance between the two species and the differences in natural habitats [27,28], we wished to explore further the THI pathway in *C*. *glabrata*.

## Results

### *C*. *glabrata* has lost the ability to synthesize PLP and HMP, leading to a defect in thiamine production relative to *S*. *cerevisiae*

To determine the alterations in the thiamine biosynthetic pathway in *C*. *glabrata* relative to *S*. *cerevisiae*, we identified the presence/absence of orthologs between the two species with regards to the known biosynthetic pathway in *S*. *cerevisiae* (Fig 1A) (gene names labeled in green are absent from *C*. *glabrata*). *C*. *glabrata* appeared to be unable to synthesize HMP, as there are no apparent orthologs of the *ScTHI5* family present in *C*. *glabrata*. Additionally, there is a lack of the *SNO*/*SNZ* genes [22] indicating that *C*. *glabrata* is unable to synthesize PLP, the precursor to HMP. Analysis of the THI regulatory pathway also indicated that *THI2* was absent from *C*. *glabrata* (Fig 1B). Through analysis of the Fungal Orthogroups and PhylomeDB websites [29,30], the pre-genome duplication common ancestor of *C*. *glabrata* and *Saccharomyces* species had the *THI5*, *SNO/SNZ*, and *THI2* genes, suggesting that *C*. *glabrata* (and the closely related “*glabrata* group” of species [22]) has specifically lost these genes relative to other species (S1 Fig). Additionally, it appears that the *Saccharomyces senso stricto* species have increased the copy numbers of the *SNO*, *SNZ*, and *THI5* genes relative to the pre-genome duplication species. Interestingly, all four of the *THI5* gene family members in *S*. *cerevisiae* are sub-telomeric and there are two copies of the three gene array *SNO/SNZ/THI5* in the genome, indicating a small scale duplication has increased the gene numbers [31]. The loss of conserved synteny of the *SNO/SNZ/THI5* gene families and the increased numbers of orthologs of these genes suggests that there was a selective advantage in the *Saccharomyces* clade to having increased thiamine and PLP biosynthesis and conversely that *C*. *glabrata* does not benefit from increased thiamine production via HMP synthesis. Recent work demonstrates that the physical linkage of genes in yeast may be an adaptation against toxic intermediates [32], and the linkage of these *SNO/SNZ/THI5* genes in *S*. *cerevisiae* may be advantageous when there is a large metabolic flux through the THI pathway.

**Fig 2:**
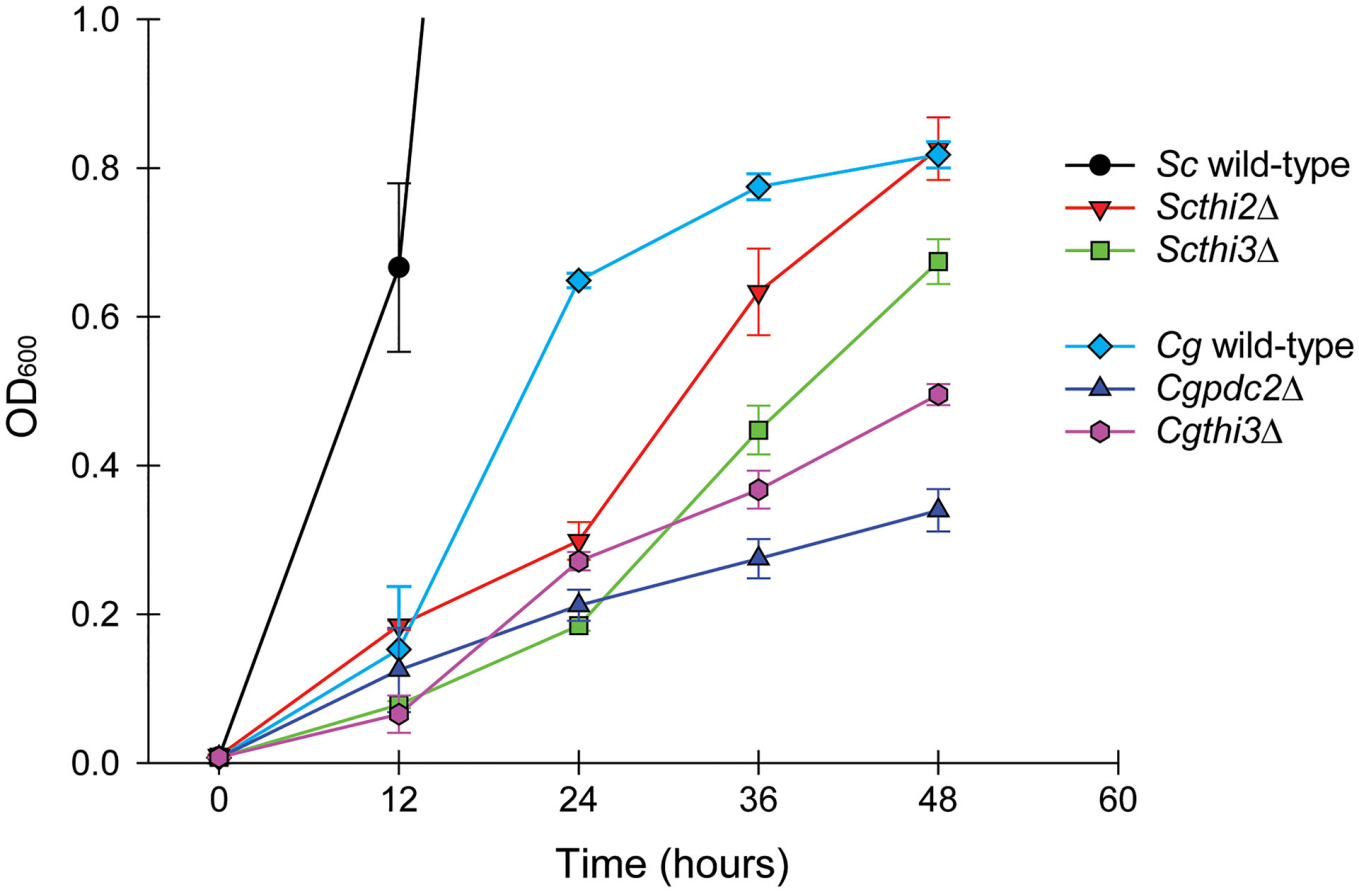
*C*. *glabrata* exhibits a growth defect relative to *S*. *cerevisiae* during thiamine starvation and utilizes the same regulatory factors. Growth during thiamine starvation of *S*. *cerevisiae* and *C*. *glabrata* wild-type strains as well as deletions of the putative regulators of the pathway: *Scthi2*Δ, *Scthi3*Δ, *Cgthi3*Δ, and *Cgpdc2*Δ. It is worth noting that in *S*. *cerevisiae*, *PDC2* is required for growth in high glucose conditions, and was not included in this analysis. Cell density (OD_600_) was measured at 12 h intervals during thiamine starvation for 48 h, and cells were diluted to remain in logarithmic growth. Error bars represent standard deviation of three experimental replicates, and this is true of all of the figures. *S*. *cerevisiae* wild-type grew to OD_600_ = 314.3±8.1 in 48 h of thiamine starvation. In high thiamine conditions, all six strains grow vigorously (OD_600_ > 300 in 48 h).

To determine whether *C*. *glabrata* utilized the THI regulatory pathway (and consequently the biosynthetic pathway), we inactivated putative regulators of the biosynthetic pathway, *THI2* and *THI3* in *S*. *cerevisiae* and *THI3* and *PDC2* in *C*. *glabrata*, to see if there was a defect in growth during thiamine starvation (Fig 2). We additionally inactivated genes in the biosynthetic pathway (S2 Fig). Deletion of *ScPDC2* was not included in this analysis because it is essential for growth in high glucose conditions [16]. *Cg*Pdc2 appears to have a different transcriptional specificity relative to *Sc*Pdc2 as deletion of *CgPDC2* does not result in an obvious growth defect in high glucose conditions (data not shown). As expected, there was a defect for the *S*. *cerevisiae* deletion strains relative to wild-type in the absence of thiamine. We also observed a small, but marked, defect in the ability of the *C*. *glabrata* mutants to grow relative to wild-type. The most parsimonious explanation for these data are that the thiamine biosynthetic pathway, while missing components, is still active in synthesizing additional thiamine, albeit at a much lower level than *S*. *cerevisiae*, and loss of either the transcriptional regulators or the biosynthetic genes leads to a defect in growth in the absence of thiamine.

**Fig 3:**
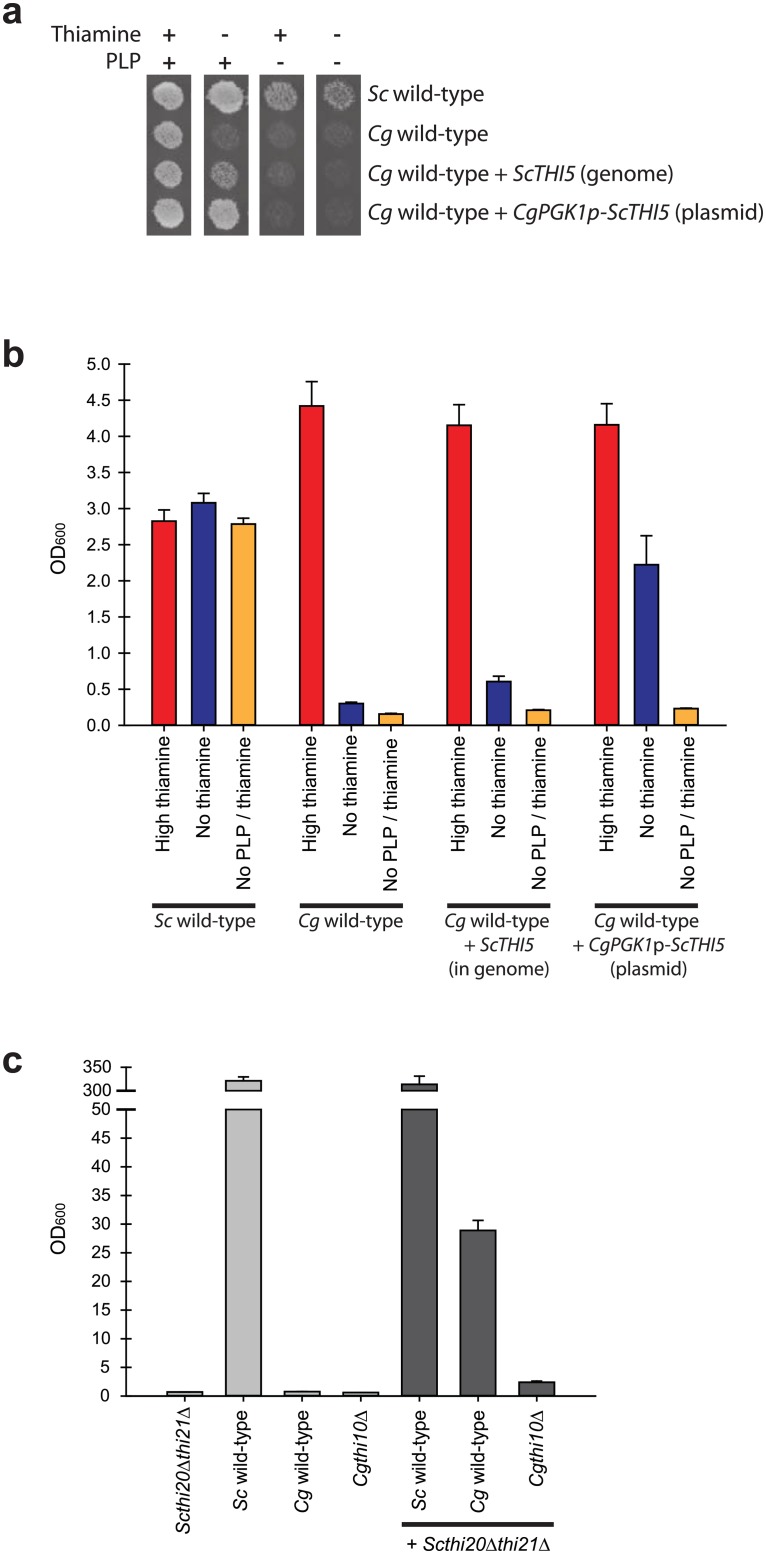
Enabling *C*. *glabrata* to make or use HMP allows for synthesis of thiamine and growth in starvation conditions. (A) Adding back *THI5* in the genome or on a plasmid restores growth to *C*. *glabrata* in thiamine starvation. Strains were grown in replete or thiamine- and PLP-starved conditions on solid agar medium. (B) The same strains as in (A) were also grown in liquid medium and cell density (OD_600_) was measured after 24 h. (C) Co-culture of *Scthi20*Δ*Scthi21*Δ strain with *C*. *glabrata* allows for vigorous growth, suggesting that *S*. *cerevisiae* supplies HMP to *C*. *glabrata* and this uptake of HMP is decreased in a *Cgthi10*Δ. Co-culture was required because in our hands HMP was not stable enough for conditioned medium experiments. Strains were grown either alone or in co-culture in thiamine starved conditions and cell density (OD_600_) was measured after 48 h. Cells were diluted over the 48 h to remain in logarithmic growth.

As the sequenced genome of *C*. *glabrata* has lost genes related to PLP synthesis, we expected this species to be a PLP auxotroph in addition to a thiamine auxotroph [22,33]. When grown in the absence of thiamine and/or PLP, *C*. *glabrata* exhibits a large growth defect whereas *S*. *cerevisiae* grows well (Fig 3A and 3B). We predicted that if *C*. *glabrata* was able to synthesize HMP, growth would be enhanced in the absence of thiamine but not in the absence of PLP, as PLP is required for metabolic reactions independent of thiamine. To test this hypothesis, we generated a *C*. *glabrata* strain that had the *ScTHI5* promoter and ORF integrated into the *CgURA3* locus. We also generated a *C*. *glabrata* strain containing a plasmid where the *ScTHI5* gene was overexpressed by the *CgPGK1* promoter, which is a highly expressed gene in standard medium conditions. We anticipated that the two promoter constructs should provide a dose-response of low and high levels of *Sc*Thi5. Growth was measured for these strains, and wild-type strains, in replete, thiamine starved, PLP starved, and thiamine/PLP starved conditions (Fig 3A and 3B). The data suggest that when a source of HMP is provided (addition of the *ScTHI5* gene), *C*. *glabrata* is able to utilize this source of HMP to generate more thiamine and allow for better growth. Additionally, a higher level of expression of *ScTHI5* through the *CgPGK1* promoter allows for even better growth in the absence of thiamine. This growth is not present when the cells are also starved of PLP, indicating that *C*. *glabrata* is actually synthesizing HMP using PLP as a precursor.

To confirm *C*. *glabrata* was utilizing HMP as a substrate for growth, we incubated *C*. *glabrata* with another source of HMP–*Scthi20*Δ*Scthi21*Δ cells. An overshoot phenomenon has been demonstrated in *S*. *cerevisiae* when too much HMP is present, resulting in an efflux of HMP from the cell. It has also been shown in *S*. *cerevisiae* that HMP can be brought into the cell when present in the environment [17]. We hypothesized that co-culturing *C*. *glabrata* with cells that are unable to make thiamine, but accumulate HMP would allow *C*. *glabrata* to grow more vigorously during thiamine starvation because *C*. *glabrata* could transport HMP into the cell and convert it into thiamine. As shown in Fig 3C, co-culturing *C*. *glabrata* wild-type with *Scthi20*Δ*Scthi21*Δ cells allows for a large increase in cell density. We also deleted the thiamine transporter, *THI10*, to determine if this gene had a role in *C*. *glabrata* obtaining HMP from *S*. *cerevisiae*. When co-cultured with *Scthi20*Δ*Scthi21*Δ cells, there was little growth for *Cgthi10*Δ, indicating that *THI10* may be involved in bringing environmental HMP into *C*. *glabrata* cells, allowing for additional thiamine synthesis. Finally, we confirmed that the *Scthi20*Δ*Scthi21*Δ cells were contributing a metabolite related to thiamine (likely HMP) because we observed very little growth when *Scthi20*Δ*Scthi21*Δ cells were co-cultured with a *Cgthi3*Δ, *Cgpdc2*Δ, or a *Cgthi20*Δ strain (data not shown).

### HPLC analysis of thiamine vitamers in both species

**Fig 4:**
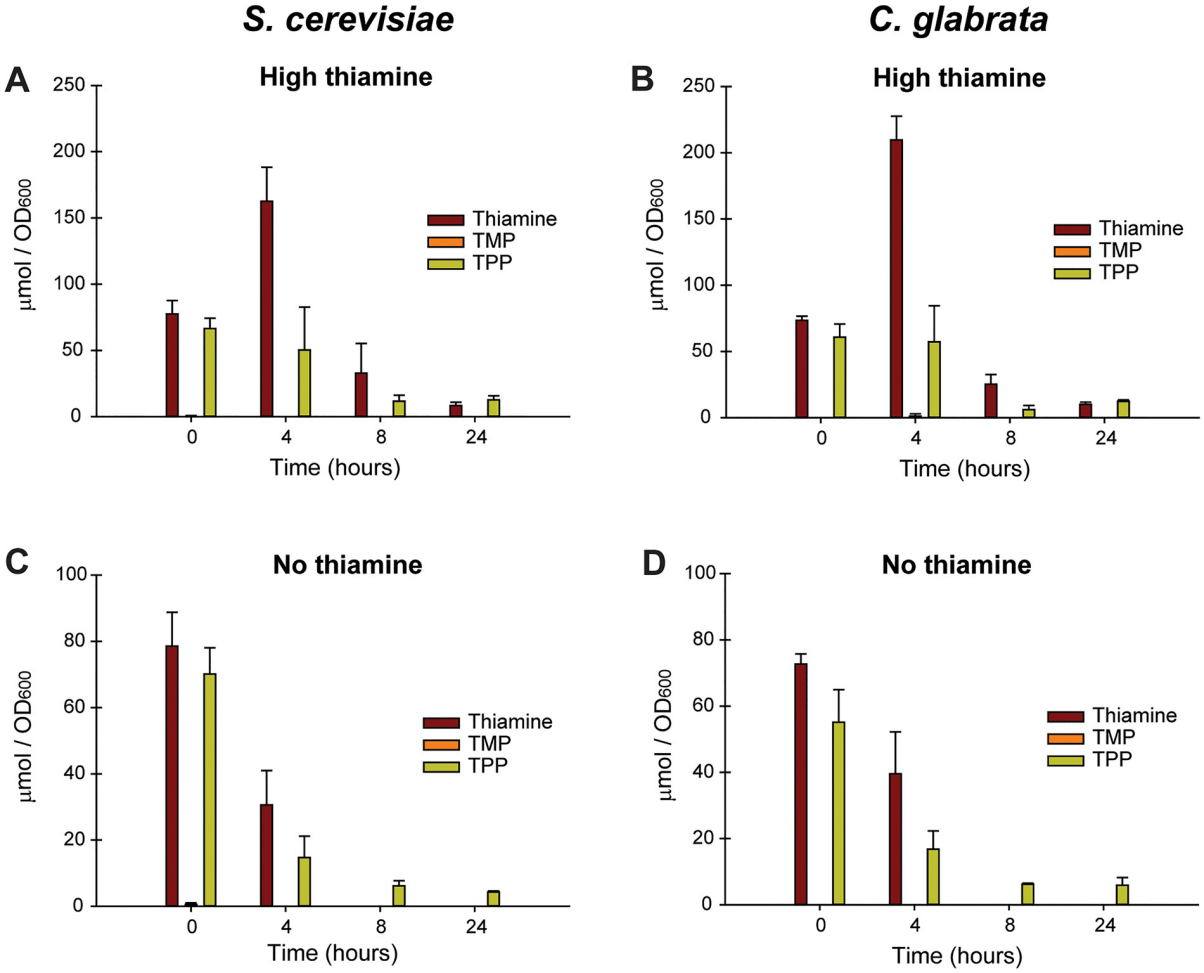
Vitamers of thiamine in *S*. *cerevisiae* and *C*. *glabrata* as detected by HPLC. (A) Wild-type *S*. *cerevisiae* and (B) *C*. *glabrata* cells grown in SD medium with sufficient thiamine. (C) and (D) are the same cells grown in SD medium lacking thiamine. Cells were inoculated at an OD_600_ = 0.1 and allowed to grow 24 h. Five OD_600_ units were harvested at given times, washed three times, and frozen at -80°C for analysis. When converted to amount per cell, *S*. *cerevisiae* contains approximately 4 pmol of TPP per cell, and *C*. *glabrata* contains 2 pmol of TPP per cell when starved for 24 h. *C*. *glabrata* cells contain approximately half the volume of *S*. *cerevisiae* cells.

We wanted to discover if the two species utilize or store thiamine in different ways, which might uncover different thiamine utilization strategies. We measured the concentration of thiamine, TMP, and TPP in both species when grown in high and no environmental thiamine conditions, using an HPLC assay. We first verified that this assay was capable of detecting the three vitamers in cell extracts (S3 Fig) and was linear for measuring thiamine vitamer concentrations (S4 Fig). We then measured the vitamer concentration on a per biomass basis (estimated by OD_600_) for both species. *S*. *cerevisiae* and *C*. *glabrata* store approximately the same amount of thiamine and TPP during growth in high thiamine (Fig 4A and 4B). Because cells are grown at a relatively high density, we are also able to observe that concentrations of vitamers decrease over growth time, which is correlated with the diauxic shift (shifting from glucose fermentation to respiration). This is suggestive that fermentation requires more TPP than oxidative phosphorylation, but additional experiments are required to validate this hypothesis. Additionally, we observed that both species shift their total vitamer concentrations to TPP during thiamine starvation which is not surprising given that TPP is the only metabolically active vitamer (Fig 4C and 4D). Finally, we observed that cells do not maintain reserves of TMP suggesting that any TMP that is synthesized is rapidly dephosphorylated and pyrophosphorylated or simply dephosphorylated.

### *C*. *glabrata* has compensated for the loss of a key transcriptional regulator of thiamine production relative to *S*. *cerevisiae*

**Fig 5:**
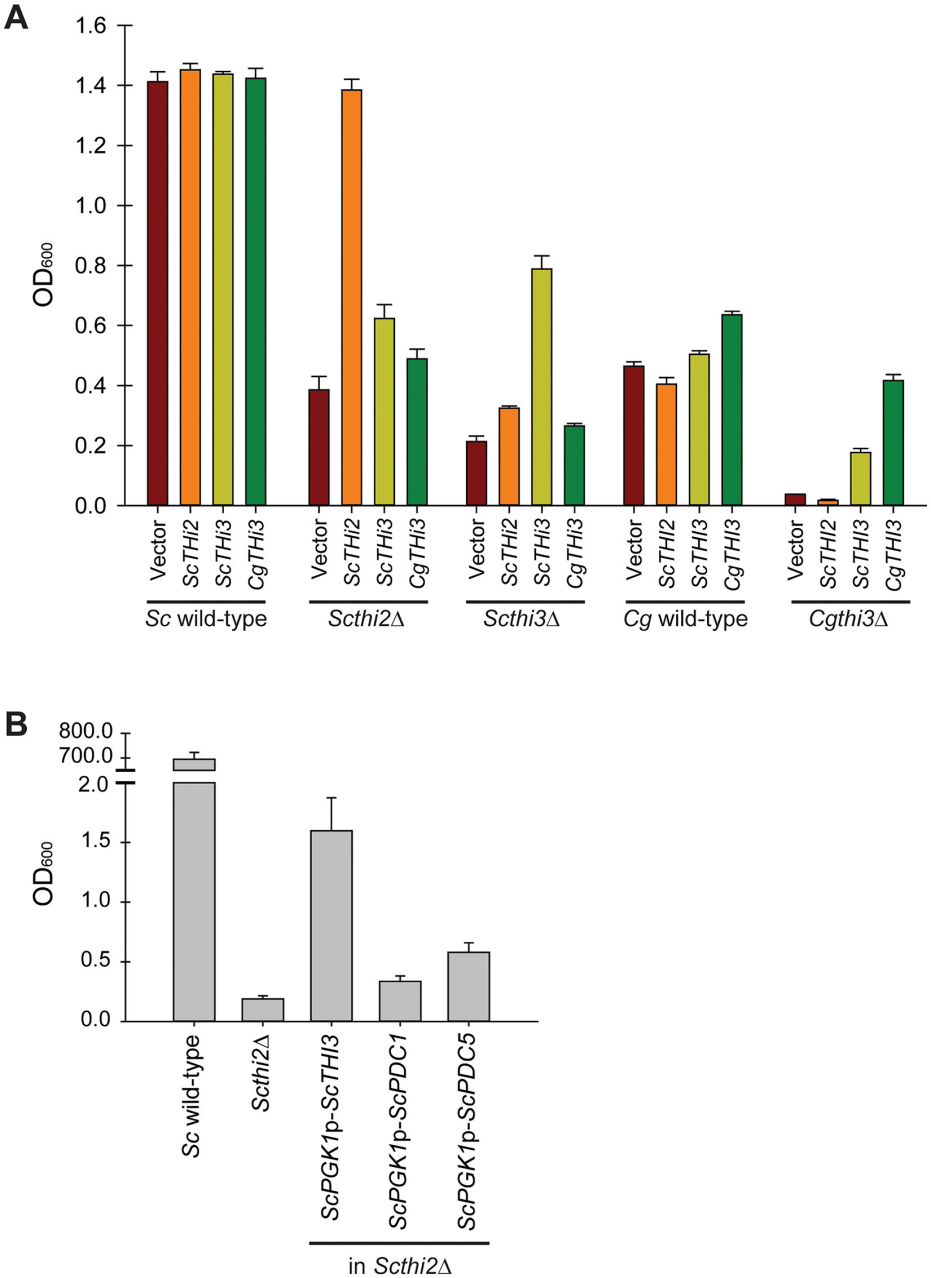
Both *ScTHI3* and *CgTHI3* are capable of suppressing the thiamine auxotrophy of the *Scthi2*Δ strain, indicating that overexpression of *THI3* is sufficient to suppress the loss of *THI2*. (A) Plasmids containing no gene (Vector), *ScTHI2*, *ScTHI3*, or *CgTHI3* were transformed into deletion strains and cell density (OD_600_) was measured after 24 h of thiamine starvation to determine if the genes could suppress the auxotrophy. (B) The highly expressed *ScPGK1* promoter was inserted through homologous recombination [35] in front of genes in the genome of a *Scthi2*Δ strain, grown in thiamine depleted conditions, and cell density (OD_600_) was measured after 72 h. Cells were diluted over the 72 h to remain in logarithmic growth.

Because *C*. *glabrata* is lacking a *THI2* ortholog, and a *Scthi2*Δ strain is a thiamine auxotroph, we wanted to understand how *C*. *glabrata* maintains any thiamine biosynthesis independent of *THI2*. We screened a *C*. *glabrata* genomic library [34] for clones that were able to suppress the thiamine auxotrophy of a *Scthi2*Δ strain, and recovered five plasmids all containing *CgTHI3*. No other plasmids reproducibly suppressed the *Scthi2*Δ auxotrophy as assayed by colony formation on solid medium lacking thiamine (data not shown). This raised the question of whether there was a function specific to *CgTHI3* or if simply expressing *THI3* from either species on a plasmid (and increasing the copy number) was capable of suppressing the *Scthi2*Δ thiamine auxotrophy. To differentiate between these two possibilities we cloned *ScTHI2*, *ScTHI3*, and *CgTHI3* (promoter and ORF) into a *HIS3* plasmid and determined the ability of each plasmid to suppress the thiamine auxotrophy of the *thi2*Δ and *thi3*Δ strains in both species (Fig 5A). All of the plasmids were functional, as they were able to complement deletion of the appropriate gene. Furthermore, both *ScTHI3* and *CgTHI3* were capable of partially suppressing the thiamine auxotrophy of the *Scthi2*Δ strain (p = 0.003 and p = 0.03 respectively by Student’s *t*-test), indicating that simple overexpression of *THI3* is sufficient to suppress the loss of the *THI2* and there is no apparent neofunctionalization of *CgTHI3* relative to *ScTHI3*.

These cross-complementation studies indicated that we should be able to overexpress *ScTHI3* in the genome of *S*. *cerevisiae* and partially suppress the thiamine auxotrophy of the *Scthi2*Δ strain. To test this, we engineered in the genome a high level promoter (*ScPGK1*) driving the expression of *ScTHI3*, which has homology to pyruvate decarboxylase genes but does not appear to have pyruvate decarboxylase activity, and two related homologs, *ScPDC1* and *ScPDC5* (49% and 50% identical to *ScTHI3*, respectively), not thought to be involved in the transcription of THI genes (Fig 5B) [16]. Overexpression of *ScTHI3* led to increased growth of the *Scthi2*Δ strain in thiamine starvation and the two non-transcriptional regulators appeared to only minimally stimulate growth in the absence of thiamine. These data suggest that high levels of the Thi3 transcriptional regulator specifically can confer the ability to grow better in thiamine starvation conditions in the absence of *THI2*. Additionally, these data suggest that *C*. *glabrata* behaves similar to a *S*. *cerevisiae* strain that is deleted for *THI2* but over expresses *THI3*.

### *C*. *glabrata* induces a different subset of THI genes to optimize uptake and minimize thiamine biosynthesis relative to *S*. *cerevisiae*

**Fig 6:**
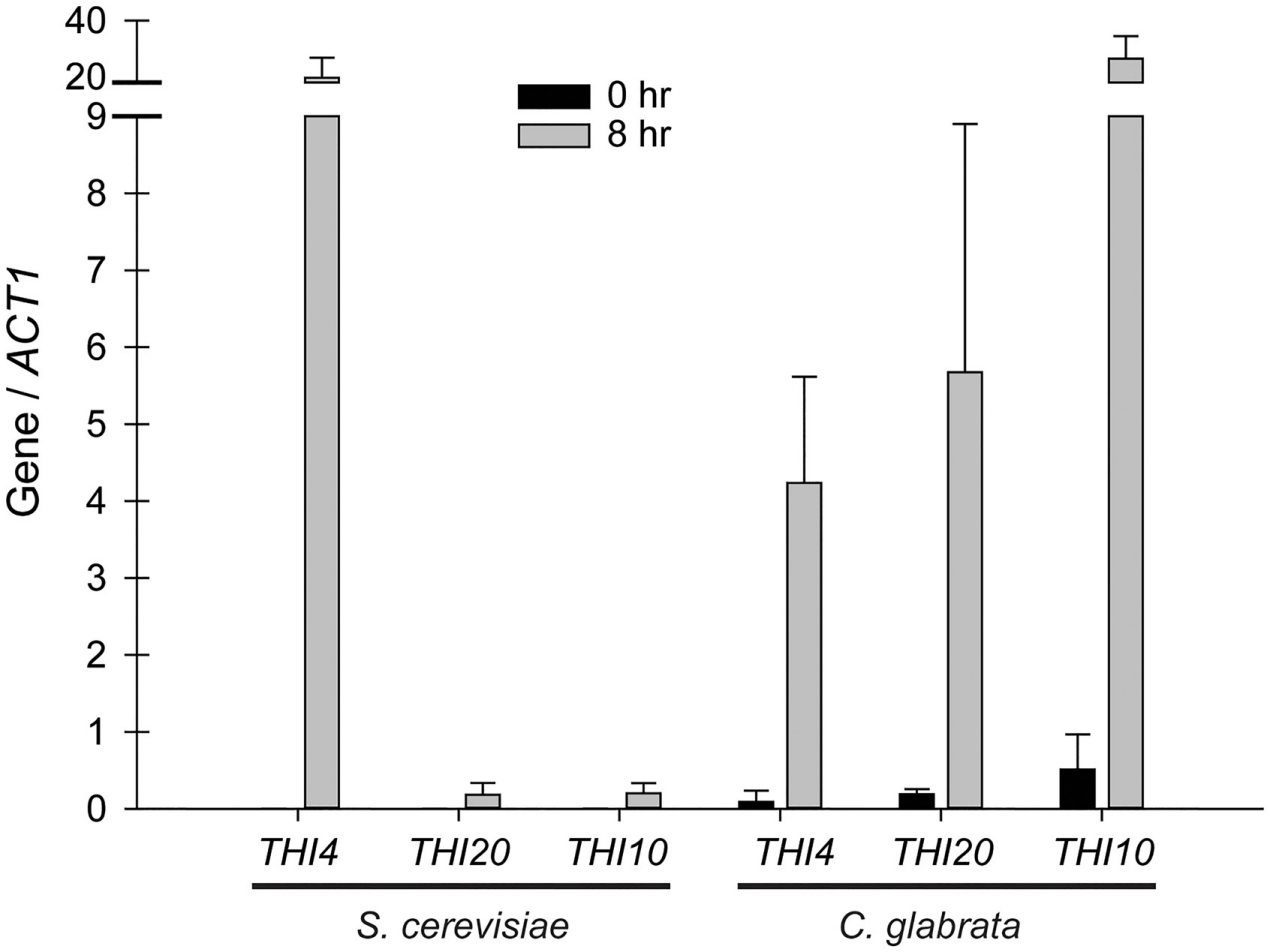
*C*. *glabrata* induces a different subset of *THI* genes to optimize uptake and minimize thiamine biosynthesis relative to *S*. *cerevisiae*. rt-qPCR analysis of *THI4*, *THI20*, and *THI10* in both *S*. *cerevisiae* and *C*. *glabrata* wild-type strains grown in thiamine depleted conditions. All expression is relative to the *ACT1* gene of the respective strain.

Because *C*. *glabrata* appears to be capable of utilizing an environmental source of HMP for thiamine biosynthesis (Fig 3), we hypothesized that the transcriptional derepression of the THI genes may have different characteristics between the two species. For example, *C*. *glabrata* might induce scavenging genes to a higher level than synthesis genes relative to *S*. *cerevisiae*. To test this, we performed rt-qPCR on candidate THI genes. We chose genes that are involved in the synthesis of the HET precursor (*THI4*), HMP phosphorylation (*THI20*), and transport of thiamine into the cell (*THI10*). The rt-qPCR analyses suggest that *S*. *cerevisiae* synthesizes thiamine *de novo* when thiamine is not present by expressing *THI4* at high levels while inducing *THI10* expression much less (Fig 6). *C*. *glabrata*, on the other hand, may be obtaining thiamine directly from the environment and does so by expressing *THI10* to high levels in an effort to uptake thiamine and HMP. A caveat to this analysis is that all of the genes are upregulated during thiamine starvation and post transcriptional mechanisms are not being considered.

### Growth characteristics of *S*. *cerevisiae* and *C*. *glabrata* are altered in replete and thiamine-starved conditions

**Fig 7:**
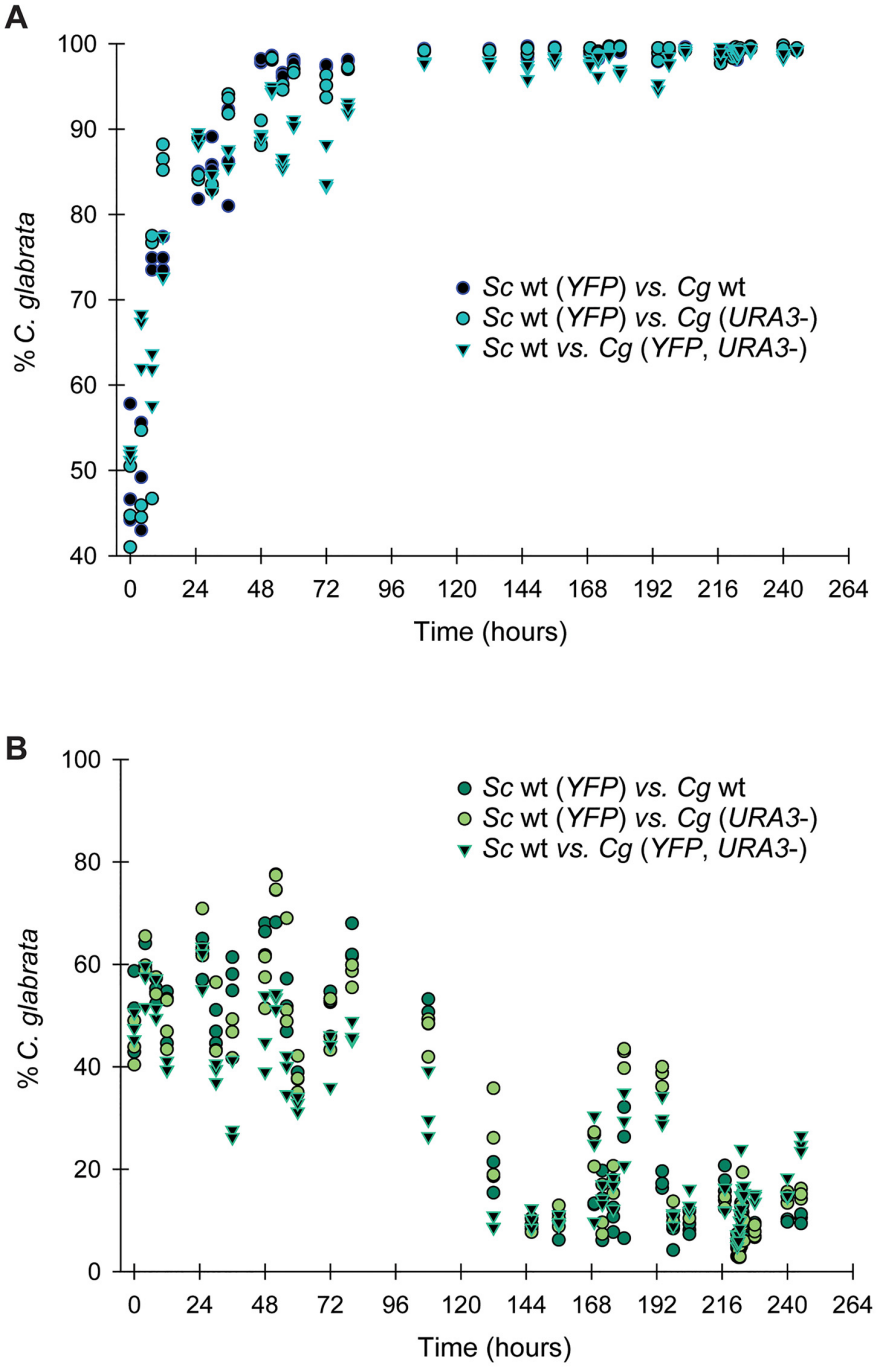
*C*. *glabrata* out competes *S*. *cerevisiae* in the presence of thiamine but not in the absence of thiamine. (A) Using flow cytometry, the percent of cells that was either *S*. *cerevisiae* or *C*. *glabrata* was determined and plotted based on the time of the measurement. Cells were maintained in a logarithmic growth phase in SD medium containing thiamine for the entirety of the assay. The data presented are from nine separate competition assays with three different sets of strains. Because the *CgADH1p-YFP* was integrated into the *CgURA3* locus, we confirmed that the presence or absence of uracil and the *URA3* gene did not affect the results. The *S*. *cerevisiae* strain was from [36]. (B) The same assay as part (A) was performed but in the absence of thiamine.

To determine the consequences of having a partial decay of the THI biosynthetic and regulatory pathway in *C*. *glabrata* relative to *S*. *cerevisiae*, we utilized co-culture conditions of both species where one species expressed a yellow fluorescent protein (YFP). This experimental setup allowed for real-time measurement of cell numbers of each species by flow cytometry. We measured cell growth of wild-type *C*. *glabrata* and *S*.*cerevisiae* during exponential growth, and regardless of auxotrophic markers or presence of YFP, determined that *C*. *glabrata* outcompetes *S*. *cerevisiae* in medium containing thiamine (Fig 7A). This was not surprising as the doubling time of *C*. *glabrata* (4.06 h ± 0.06) is faster that *S*. *cerevisiae* (4.44 h ± 0.23) in synthetic medium when grown individually. Within three days, over 95% of the cells in the co-culture are *C*. *glabrata*.

During growth in medium lacking thiamine, we observed more complex growth characteristics, but they are consistent with *C*. *glabrata* not responding to thiamine starvation as efficiently as *S*. *cerevisiae* (Fig 7B). We observe consistent short-term advantages of *C*. *glabrata* every time cultures are diluted. We do not know why this is the case, but could be a consequence of *C*. *glabrata* being able to grow better in fresh medium and grow more rapidly than *S*. *cerevisiae* for a short period. In the long-term though, *C*. *glabrata* is unable to synthesize enough thiamine for robust growth and so *S*. *cerevisiae* slowly dominates the co-culture. Interestingly, we observe a phenomenon similar to frequency-dependent selection in co-culture in both high and no thiamine conditions; the less fit species is still maintained (between 1–5%) after 10 days of culturing, even though the doubling times of individual cultures suggest that one species should completely outcompete the other. We believe that this indicates that in both conditions, there are trade-offs with regards to growth between the two species and potentially compounds can be transferred between species to allow both species to grow more vigorously.

## Discussion

We demonstrate a partial decay of the THI biosynthetic pathway in *C*. *glabrata* that is a derived trait in the *C*. *glabrata* clade relative to closely related species [22]. Comparison of the THI pathways of *S*. *cerevisiae* and *C*. *glabrata* have allowed for a number of observations that suggest the two species thrive in different niches. *S*. *cerevisiae* grows on decaying plant material and *C*. *glabrata* grows on mammalian mucosa [37,38]. Of the 139 orthologs absent in *C*. *glabrata* relative to *S*. *cerevisiae*, at least nine are involved in PLP (vitamin B_6_) and thiamine (vitamin B_1_) biosynthesis [31]. PLP participates in core metabolism independent of thiamine metabolism, and thus, both vitamins are required for proper growth. Our results suggest that mammalian tissue is supplying PLP and HMP/thiamine, and that *S*. *cerevisiae* may not derive those compounds as readily from decomposing plant material. It is worth highlighting that it is not clear what the “natural” environment of the two species is [28,39], but given that *C*. *glabrata* is isolated from human GI tract and *S*. *cerevisiae* is often isolated from rotting plant material, our assumption is not unreasonable.

Whereas the HMP part of the thiamine biosynthetic pathway has decayed in *C*. *glabrata*, it is noteworthy that the transcriptional wiring has also changed between the two species. *A priori* there is no apparent need to lose *THI2* just from the *glabrata* clade of species, given that all of the other recent common ancestors contain *THI2*. However, this event is similar to the loss of the absolute requirement for *CgPHO2* in the phosphate signal transduction pathway [40], suggesting that *C*. *glabrata* (and related species) has simplified its transcriptional response in at least two signal transduction pathways. Further work is required to determine whether *S*. *cerevisiae* has modified the core THI pathway for the purposes of generating multiple transcriptional outputs or if *C*. *glabrata* has streamlined its genome. *S*. *cerevisiae* has amplified the *ScTHI5* family relative to most species possibly indicating that the amount of thiamine required by *S*. *cerevisiae* is larger in its environment than *C*. *glabrata*. Interestingly, Nosaka *et al*. has demonstrated that some THI promoters in *S*. *cerevisiae* (*ScTHI2* and *ScTHI10*) are not dependent on *ScTHI2*, suggesting that *Sc*Thi2p might be an accessory transcription factor that allows for an increased level of expression of the THI transcripts [4,41]. The difference in specificity of the Pdc2 transcription factor also underlies the transcriptional rewiring; *Sc*Pdc2 is critical for the regulation of pyruvate decarboxylase genes and this is not true of *Cg*Pdc2 [16].

Our measurements of thiamine vitamer concentrations by HPLC also indicate that the amount of thiamine per biomass is varied depending on condition—i.e. during periods of slower growth there is apparently less need for TPP per cell. This may indicate that *S*. *cerevisiae* experiences times in its environment where the ability to synthesize a large amount of thiamine is beneficial. For example, during fermentation it is likely that there is large flux of glucose through the TPP requiring enzyme pyruvate decarboxylase, whereas during slower growing conditions (potentially growing on mucosal membranes) there is less TPP needed for growth as respiration is much more efficient at energy production. The expansion of the *SNO*/*SNZ*/*THI5* gene families in the *Saccharomyces* clade is consistent with this argument. It is worth noting that pathogenic fungi, including *C*. *albicans*, have an apparently fully functioning THI pathway, indicating that the partial loss of the THI pathway that we observe in *C*. *glabrata* is not a requirement for pathogenicity. Likewise, closely related species to *C*. *glabrata* that do not appear to be commensal with mammals have similar THI pathways to *C*. *glabrata*–i.e. the same losses of genes (S1 Fig and [22]). This loss of genes may be a function of subtly different growth niches that remain to be understood more fully. Our work is reminiscent of work with biotin biosynthesis in yeast species, where some *Saccharomyces* species have lost the most upstream parts of the biosynthetic pathway [42]. Interestingly, in that example, selection pressure appears to have led to horizontal gene transfer from bacteria of the upstream genes.

Finally, the characterization of the THI pathway in *C*. *glabrata* has uncovered potential future avenues of antifungal research. As current pharmacological agents in the chloroquine family have been demonstrated to target thiamine uptake in yeast and mammalian cells [43], targeting thiamine biosynthesis and uptake may be a good drug target for treatment of *C*. *glabrata* infections.

## Methods

### Yeast strains

Yeast strains used in this study are listed in Table 1. Primers used to make these strains are listed in S1 Table. Deletion mutants were generated using antibiotic resistance genes *KANMX6* or *NATMX6* and homologous recombination to precisely delete ORFs [40,44]. Deletion was confirmed by PCR. Genes were incorporated into the genome by homologous recombination to precisely replace *URA3*, selected with 5-FOA, and confirmed by PCR.

**Table 1:** Strains used in this study.

***S*. *cerevisiae* strains**	
DC3	*S*. *cerevisiae* wild-type K699 *ade2-1 trp1-1 can1-100 leu2-3*,*112 his3-11*,*15 ura3*
DC10	*Scleu2*::*ScURA3-KANMX6-ScADH1pr-YFP* in DC3 [41]
DC126	*Scthi2*Δ*KANMX6* in DC3
DC143	*Scthi3*Δ*NATMX6* in DC3
DC169	*Scthi20*Δ*NATMX6 Scthi21*Δ*KANMX6* in DC3
DC170	*ScPDC1pr*::*HIS3*^+^*-ScPGK1pr-ScPDC1* in DC126
DC171	*ScPDC5pr*::*HIS3*^+^*-ScPGK1pr-ScPDC5* in DC126
DC172	*ScTHI3pr*::*HIS3*^+^*-ScPGK1pr-ScTHI3* in DC126
***C*. *glabrata* strains**	
DG5	*C*. *glabrata* wild-type (*his3*^-^) (BG99) [45]
DG141	*Cgthi3*Δ*NATMX6* in DG5
DG155	*Cgthi4*Δ*NATMX6* in DG5
DG176	*C*. *glabrata* wild-type (*his3*^-^ *ura3*Δ*NATMX6*)
DG226	*Cgura3*Δ*ScTHI5* in DG5
DG234	*Cgura3*Δ*CgADH1pr-YFP* in DG5
DG266	*Cgthi10*Δ*NATMX6* in DG5
DG271	*Cgpdc2*Δ*NATMX6* in DG5
DG277	*Cgthi20*Δ*NATMX6* in DG176

To construct plasmids containing genes, the promoter and the ORF of the gene were amplified (primers in S1 Table) and cloned by homologous recombination into a pRS313 (*HIS3*) vector [46].

### Growth conditions

Yeast strains were grown in YEPD medium or synthetic dextrose (SD) medium with complete supplement mixture (CSM), either with or without histidine (Sunrise Science Products; San Diego). For induction of *THI* promoters and genes, strains were grown at 30° to logarithmic growth phase (OD_600_ ~0.2–0.5) in SD medium with thiamine. Cells were harvested by centrifugation, washed three times with medium lacking thiamine, and transferred to SD medium with thiamine (high thiamine), without thiamine (no thiamine), or without PLP and thiamine (No PLP/thiamine) at a starting OD_600_ of 0.05 in 5 mL. Strains were grown in these conditions from 24 to 72 h to measure cell density (OD_600_). During growth, cells were diluted to remain in logarithmic growth. For quantitative reverse-transcription PCR analysis, strains were grown for 8 h in these conditions.

### Bioinformatic Analysis of the *THI* pathway in *C*. *glabrata*

Orthologs of the *S*. *cerevisiae THI* genes were identified in yeast species using the Fungal Orthogroups, PhylomeDB, and YGOB websites [29,30,47] as well as the Saccharomyces Genome Database [48] and the Candida Genome Database [49]. For generation of S1 Fig, orthogroups were identified from reference [29,30] and a variety of pre- and post-genome duplication species were chosen to identify trends in the number of copies of genes in each family.

### Growth in co-culture

After washing the cells three times in medium lacking thiamine, the volume of each culture needed to inoculate at a starting OD_600_ of 0.02 for *S*. *cerevisiae* and 0.01 for *C*. *glabrata* in 5 mL was calculated. This difference in OD_600_ is derived from a comparison of OD_600_ to cell count of the two species by flow cytometry (data not shown), which found that there is twice the number of *C*. *glabrata* cells per O.D. as compared to *S*. *cerevisiae* cells; *C*. *glabrata* cells are smaller than *S*. *cerevisiae*.

### HPLC analysis

The methods to analyze the vitamer concentrations are adapted largely from [50]. Cultures were inoculated at OD_600_ = 0.1 at t = 0 and grew to an OD_600_ > 3 after 24 h. The vitamers were extracted from the yeast cells (each sample = 5 OD_600_) through bead beating in 0.1 M HClO_4_, adapted from methods in [51]. Samples for the calibration curve were dissolved in 0.1 M HCl and diluted in 0.1 M HClO_4_ to inject 806, 605, 403, 201, and 81 pmol of each vitamer. To track vitamers, the samples were oxidized with 30 mM K_3_[Fe(CN)_6_] to allow for fluorescence detection of the vitamers at 366 nm excitation and 436 nm emission. Before subjecting samples to HPLC, samples were filtered with a 0.2 μm syringe filter and 10 μL was injected on a RP-amide C16 column (Supelco) with 80% 0.4 M phosphate/20% methanol as the isocratic mobile phase at 1 mL/min for a 10 min sample analysis time [50]. Calibration curves were analyzed each day at the beginning and end of runs to account for any changes in instrument sensitivity. Approximate retention times are thiamine at 7.3 min, TMP at 4.2 min, and TPP at 3.1 min. To calculate concentration of the unknown samples, the integration area of the samples was converted into pmol of vitamer based on appropriate calibration curves and then normalized to OD_600_.

### Identification of *C*. *glabrata THI2* analog

To screen for the *C*. *glabrata THI2* analog, a *C*. *glabrata* genomic DNA library [34] was transformed into a *Scthi2*Δ strain. Approximately 16,500 transformants were plated and then replica plated onto SD plates lacking uracil and thiamine to identify clones that were able to suppress the thiamine auxotrophy of the *Scthi2*Δ strain. Five plasmids were recovered using a yeast plasmid DNA purification protocol and transformation into *Escherichia coli* cells. The plasmids were then sequenced and all 5 contained DNA near the *CgTHI3* gene [24].

### Quantitative reverse-transcription PCR (rt-qPCR)

RNA was extracted using a standard phenol—chloroform protocol [52] and converted to cDNA by a reverse-transcription reaction (Bio-Rad iScript cDNA synthesis kit). Quantitative PCR was performed with a CFX qPCR machine (Bio-Rad) using SybrGreen I dye in a 50-μL reaction. Primers were designed for *S*. *cerevisiae* and *C*. *glabrata THI* genes *THI4*, *THI20*, *THI10*, and *ACT1* (primers in S1 Table). The amount of transcript for each gene was normalized to *ACT1*, which does not change expression in differing thiamine conditions. Each gene was also amplified using 10-fold genomic DNA dilutions as an amplification control.

### Co-culture competition assay

Each of the strains were grown overnight in nutrient rich medium and inoculated at starting OD_600_ of 0.002 for *S*. *cerevisiae* strains and 0.001 for *C*. *glabrata strains* in 5mL of media in triplicate in either high or no thiamine media. The mixtures were subjected to flow cytometry (Accuri C6, BD biosciences) at time-points 0, 4, 8, 12, and 24 h after the initial pipetting. Every 24 h the mixture was back-diluted into 5 mL of medium to an OD_600_ of 0.003. Flow cytometry allowed for the absolute number of cells to be counted as well as species based on which species contained YFP. We confirmed that the *ADH1pr-YFP* constructs did not alter the viability of the strains by competing them with the appropriate parental strain for 100 h.

## Supporting Information

S1 Fig Presence, absence, and number of homologs of each gene in the THI pathway in a diversity of Ascomycetes. Using YGOB, PhylomeDB, and Fungal Orthogroups [29–31], we identified likely orthologs and paralogs of the *S*. *cerevisiae* genes involved in thiamine biosynthesis, uptake, and regulation of the biosynthetic pathway. We have highlighted in red genes where the number is in conflict through the different analyses. The species are presented as a consensus phylogeny combining the work of [22,29]. Shading of the table is based on the number of homologs to highlight where we believe there has been gain/loss of genes in the THI pathways of specific clades.
(EPS)

S2 Fig Growth of various *C*. *glabrata* thiamine biosynthetic mutants in medium lacking thiamine. Growth during thiamine starvation of *C*. *glabrata* wild-type as well as strains where thiamine biosynthetic and regulatory genes are deleted: *Cgpdc2*Δ, *Cgthi3*Δ, *Cgthi4*Δ, *Cgthi20*Δ, and *Cgthi10*Δ. Cell density (OD_600_) was measured at 24 h intervals during thiamine starvation for 48 h, and cells were diluted to remain in logarithmic growth. Error bars represent standard deviation of three experimental replicates. In high thiamine conditions, all strains grow vigorously (OD_600_ > 300 in 48 h).
(EPS)

S3 Fig Example of HPLC traces of thiamine vitamers. (A) Standards of equal amounts of the three vitamers injected on HPLC: thiamine, TMP, and TPP. (B) Extract of *S*. *cerevisiae* injected on HPLC. Trace is of a single run with the fluorescence detection set at 436 nm. (C) Extract of *C*. *glabrata* injected on HPLC.
(EPS)

S4 Fig Sample calibration curve of thiamine vitamers. The graph is of the amount of vitamer injected vs. integrated area of peak.
(EPS)

S1 Table Primers used in this study. (DOCX)
