# Supplementary material for: Partial Decay of Thiamine Signal Transduction Pathway Alters Growth Properties of Candida glabrata
Source: PLoS One. 2016 Mar 25;11(3):e0152042. doi: 10.1371/journal.pone.0152042 (PMC4807840; doi:10.1371/journal.pone.0152042)
Supplement: S1 Table — (DOCX) [file pone.0152042.s005.docx]

Supplementary Table 1. Primers used in this study.

| **Primers to inactivate genes** | | | |
| --- | --- | --- | --- |
| *Scthi2*Δ | O784 | GCACTAGAACCAAATGGTCAATAGTAAGAGGCAGCAGAGACGGATCCCCGGGTTAATTAA |  |
|  | O785 | ATGAGTGAAGGGAAGGCTCAATAAGCCTAGTCCTGCATGGGAATTCGAGCTCGTTTAAAC |  |
|  | O786 | GCGTCTTAGCGGCATAAATG | check primer |
| *Scthi3*Δ | O1053 | TTTGGTGTAGGCGAATTATCGGCAATCAATGGCGTGGCCGcggatccccgggttattaa |  |
|  | O1054 | GAGCACAACGTGGCATTATATATAGCATAGAAGAGATGCGgaattcgagctcgtttaaac |  |
|  | O1024 | ATGAATTCTAGCTATACACA | check primer |
|  | O1025 | TGAAATACCGTGAAATCACC | check primer |
| *Scthi20*Δ | O1293 | TTAATAAATAATATATAAGGAAAACGTCTCGCATTTTACTCGGATCCCCGGGTTAATTAA |  |
|  | O1294 | ATATCTTTATCTATTGTGCATAAGCCATCTTATCTACTCGGAATTCGAGCTCGTTTAAAC |  |
|  | O1295 | CTCCGTACTTCTTTAGCGAC | check primer |
| *Scthi21*Δ | O1296 | GGAAATATACATAAGGAAAGCAACTGTCTTGCCACAATGACGGATCCCCGGGTTAATTAA |  |
|  | O1297 | ATCGATGTGTCTATTCATTCATATTCTAGAGCGGCGGTCCGAATTCGAGCTCGTTTAAAC |  |
|  | O1298 | CACACCATGAAAGTGCACGC | check primer |
| *Cgthi3*Δ | O1015 | atgaattatactgaagaatatgggcttccaagtcagatttCGGATCCCCGGGTTAATTAA |  |
|  | O1016 | tcataaatacatttgactcctagaatgcagttcagtcgacGAATTCGAGCTCGTTTAAAC |  |
|  | O1017 | tccatagttattaaacttgagtg | check primer |
| *Cgthi10*Δ | O1612 | GTAGAAATTATACAAGCAAATCCACATACAGCCGTCAAACACGGATCCCCGGGTTAATTAA |  |
|  | O1613 | GAGCTGGAAGCTTATTCCAGGCTTTTGGATGCAAGTTTTAGGAATTCGAGCTCGTTTAAAC |  |
|  | O1614 | GGCTATGATAACTGCATAACTC | check primer |
| *Cgpdc2*Δ | O1507 | ttttggaagctggaaaaacgagctagagtgcaccgatcagCGGATCCCCGGGTTAATTAA |  |
|  | O1508 | tgaaaacgctctttgacatacataagtatatagcaagttcGAATTCGAGCTCGTTTAAAC |  |
|  | O1509 | AGTACAACTTACCACCCAACACAC | check primer |
| *Cgthi4*Δ | O1063 | aaaaacagaccagacctaaaaatcgccattctagaggccacggatccccgggttattaa |  |
|  | O1064 | ttactcagcgaagtgcttcaagatttcttcaccagctttggaattcgagctcgtttaaac |  |
|  | O1078 | atgtctgctactgctaccac | check primer |
| *Cgthi20*Δ | O1706 | CTTCAATCAATTGCACACCATAGCTCGGAGAACTTACAATCGGATCCCCGGGTTAATTAA |  |
|  | O1707 | TCTATCTATGTCTATAAGTGTAAAGGAATTTAATTTCCCTGAATTCGAGCTCGTTTAAAC |  |
|  | O1716 | GTCTTGAATATCGAACCATTGTGCGCTGATATTGAACACATATCTTCAATCAATTGCACA |  |
|  | O1717 | TCGTTAATCTATGTATCTCTTTATAGCACTGTATCAAAACTACTCTATCTATGTCTATAA |  |
|  | O1715 | TCAACTGAACGAAGGTCGAAC | check primer |
| *Cgura3*Δ | O563 | ctttgggtccatacatttgcttgcttaagactcatgttgacggatccccgggttaattaa |  |
|  | O564 | tcagtttcctattcttttcaagtaagcatcccagccggctGAATTCGAGCTCGTTTAAAC |  |
|  | O565 | atgtccagtgcctcatatttac | check primer |
| **Inserting the *ADH1*pr-*YFP* into the *CgURA3* locus** | | | |
| *CgADH1*pr | O1526 | ggtggcggccgctctagaactagtggatccCGGTCCTAGTGTTCATACTG | These two primers place the *CgADH1*pr in front of *YFP* by gap repair. Upon cloning this plasmid the next two primers were used to insert this construct into the *CgURA3* locus. |
|  | O1527 | accagtgaataattcttcacctttagacattgtttatgtgttttttgcag |  |
| *CgADH1*pr-*YFP* | O1528 | ctttgggtccatacatttgcttgcttaagactcatgttgaCGGTCCTAGTGTTCATACTG |  |
|  | O1529 | tcagtttcctattcttttcaagtaagcatcccagccggctcgaggcaagctaaacagatc |  |
|  | O1530 | CATTGCCTCTCATATCCGTG | check primer with a *YFP* specific primer |
|  | O245 | GGGACAACACCAGTGAATAATTCTTCACC | *YFP* specific primer |
| **Placing *ScPGK1pr* in front of ORFs** | | | |
| *ScPDC1* | O1532 | ATCTTTCGAACAAATATTTACCCAAAGTAATTTCAGACATtatagttttttctccttgac |  |
|  | O1533 | GGTGATGGCACATTTTTGCATAAACCTAGCTGTCCTCGTTgaattcgagctcgtttaaac |  |
|  | O1534 | TCGTGTGATGAGGCTCGTGG | *PDC1* check with *PGK1* check |
| *ScPDC5* | O1536 | ATCTTTCAAATAAATATTTACCTAAGGTTATTTCAGACATtatagttttttctccttgac |  |
|  | O1537 | TGATTATGACAATCTCTCGAAAGAAATTTCATATGATGAGgaattcgagctcgtttaaac |  |
|  | O1538 | CAAAGGCCAAGGAAATAAAGC | *PDC5* check with *PGK1* check |
| *ScTHI3* | O1539 | ACTTCGGCAGTGCATATCTCTGTGTATAGCTAGAATTCATtatagttttttctccttgac |  |
|  | O1540 | AAATTGATGAAACTTGCGATGCATTGAACAAATTTCCCTTgaattcgagctcgtttaaac |  |
|  | O1541 | GTTTCTGCACCTTCATCGTC | *THI3* check with *PGK1* check |
|  | O1535 | gtttaaacgagctcgaattc | *PGK1* check |
| **qPCR primers** | | | |
| *ScTHI10* | O1113 | CCATTCAAAATTACTGTCAAAC |  |
|  | O1114 | AGCTTTTTCACTGGCGGTGGAGG |  |
| *ScTHI4* | O1115 | CTTGAGTCAAAAGCATGGTGTC |  |
|  | O1116 | GTTTCAAAATTTGCTCAGCAGC |  |
| *ScTHI20* | O1279 | GTTCTTCTTTAGCTGGAAAG |  |
|  | O1280 | TCACTGAGCATTTTCTCCAA |  |
| *CgTHI4* | O1119 | ggacatgaacaatgccgaagc |  |
|  | O1120 | ctcagcgaagtgcttcaagat |  |
| *CgTHI20* | O1285 | CTACATCAGGTTCTTCCCTA |  |
|  | O1286 | ATTCTGTCCAAAGGAACTTC |  |
| *CgTHI10* | O1287 | CGTGGTTGGGTGGTCTATGT |  |
|  | O1288 | ATCCAGAGTTGTTGACCGTA |  |
